# Improved matrix coating for positive- and negative-ion-mode MALDI-TOF imaging of lipids in blood vessel tissues

**DOI:** 10.1007/s00216-019-01826-x

**Published:** 2019-04-30

**Authors:** Christina Meisenbichler, Christian Doppler, David Bernhard, Thomas Müller

**Affiliations:** 1 Institute of Organic Chemistry, Leopold-Franzens University Innsbruck, 6020 Innsbruck, Austria; 2 Center for Medical Research, Medical Faculty, Johannes Kepler University Linz, 4020 Linz, Austria

**Keywords:** MALDI MSI, Matrix vapor deposition/recrystallization, Lipidomics, Thoracic aortic aneurysm

## Abstract

High-quality matrix-assisted laser desorption/ionization mass spectrometry imaging (MALDI MSI) of lipids in biological tissue relies on the fabrication of a homogeneous matrix coating featuring best possible analyte integration. This communication addresses a matrix vapor deposition/recrystallization process for the application of 1,5-diaminonaphthalene (1,5-DAN) onto slices of human aortic tissue. The matrix coating is compatible with both positive- as well as negative-ion-mode MALDI MSI facilitating a significantly enhanced detection of lipid-related signals in different cell layers of blood vessel walls.

**Electronic supplementary material:**

The online version of this article (10.1007/s00216-019-01826-x) contains supplementary material, which is available to authorized users.

**Figure:**
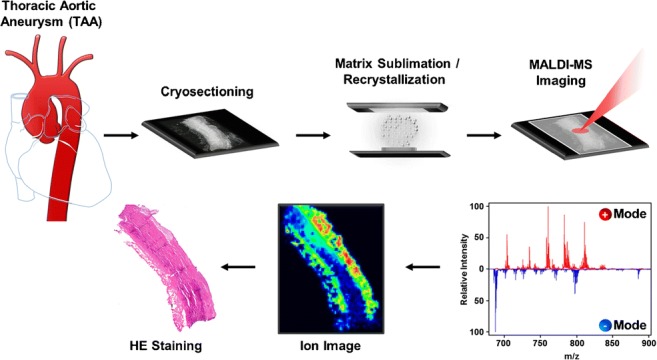
Graphical abstract

## Introduction

Mass spectrometry imaging (MSI) has become a powerful tool for the analysis of biological tissue, in particular due to its potential to monitor 2D distributions of chemicals in different tissue layers [1–3]. In matrix-assisted laser desorption/ionization mass spectrometry imaging (MALDI MSI), the fabrication of a homogeneous, analyte containing matrix coating [4] is a prerequisite for high sensitivity but also the key step for the acquisition of highly resolved data. This can be achieved by either applying a matrix in solution [5–10] or depositing solvent-free matrix onto the sample surface [11–15]. Although the application of a dissolved matrix (usually using an automated spray system [16]) features an immediate orthogonal integration of the analytes into the matrix, it involves the risk of lateral analyte delocalization, which is a clear limitation for high-resolution imaging. On the other side, matrix vapor deposition [11] produces exceptionally homogeneous coatings and precludes artifacts due to analyte diffusion [17]. Lacking integration of analytes into coating can easily be compensated by recrystallization of the matrix layer [18, 19]. Moreover, sublimation of the matrix is an additional purification step resulting in excellent low chemical noise within the MALDI mass spectrum.

Thoracic aortic aneurysms (TAA) are among the 20 most relevant causes of death in all individuals and the 17th most common in elderly patients above 65 years [20]. An exact number of cases is difficult to determine, since most aortic aneurysms are clinically silent and only around 5% of all patients are symptomatic before an acute event occurs [21]. A previous metabolome-based study provided first evidence that lipids might also be potential biomarkers for the occurrence of thoracic aortic aneurysms (TAA) [22]. In a targeted metabolomics approach, tissue concentrations of sphingomyelins and phosphatidylcholines were identified to potentially distinguish pathological from healthy tissue. In order to strengthen the significance of these findings, we utilized MSI to both localize lipids in different tissue layers as well as overcome the limits of a targeted analysis focusing on a restricted number of possible biomarker candidates.

In this study, we present a concise sample preparation method for MALDI MSI to examine the histological distribution of lipids in aortic tissue based on the matrix deposition/recrystallization methodology. Method development was focused on (i) a short sample preparation procedure, (ii) a possibility of keeping the sample cooled or under vacuum as long as possible, and (iii) enhancing sensitivity for the identification of a maximum number of lipid-related signals.

## Material and methods

### Reagents and chemicals

1,5-Diaminonaphthalene (1,5-DAN) was purchased from Sigma-Aldrich (St. Louis, USA). Isopropanol, methanol, toluene, and chloroform were purchased from VWR (Fontenay Sous Bois, France), and acetonitrile and ammonium formate from Riedel-de Haen (Seelze, Germany). Formic acid was purchased from Fluka (Buchs, Switzerland) and 0.9% saline water from Fresenius Kabi (Graz, Austria).

### Tissue sections

Frozen human aortic tissue was obtained from the Medical University of Innsbruck [22]. The human aortic tissue was stored at − 80 °C until it was cut into 8-μm-thick slices at − 20 °C by using a Leica CM 1850 cryostat (Leica Microsystems GmbH, Wetzlar, Germany). The tissue sections were thaw-mounted on 1 × 1 cm stainless steel or standard ITO slides (Bruker Daltonics) and stored again at − 80 °C until needed.

### Tissue washing step (optional)

The thaw-mounted tissue sections were covered with 6 drops of saline water or ammonium formate solution (prepared according to [23]). After 30 s (or 1.5 min in case of ammonium formate), the washing solution was removed by tilting the surface. The sections were transferred into the sublimation apparatus and dried for several minutes prior to matrix vapor deposition.

### Matrix vapor deposition

Matrix deposition was carried out using an in-house built sublimation apparatus (for details, see Fig. S2 in the Electronic Supplementary Material, ESM). An ITO glass slide with an aortic tissue section was fixed onto the cooling plate using adhesive tape. Two hundred milligrams of 1,5-DAN was placed in a glass Petri dish on top of a hot plate. After closing the apparatus, the vacuum was kept below 2 × 10^−3^ mbar using a rough pump and the hot plate was heated to a maximum temperature of 136 °C. The sample was cooled below 20 °C, while sublimation was performed within 5 min at an increasing plate temperature between 123 and 136 °C and gave a DAN coating at around 0.11 mg × cm^−2^ (see Fig. S2 in the ESM).

### Matrix recrystallization

After matrix deposition, the sublimation apparatus was opened, the excessive matrix removed, and the hot plate quickly cooled down to about 20 °C above the boiling point of the solvent used for recrystallization. A glass Petri dish was filled with 5 mL of acetonitrile, chloroform, or toluene and put on the hot plate. The apparatus was then closed for 30 s in order to expose the matrix coating of the sample to the vapor of the boiling solvent (see Fig. S2 in the ESM).

### MALDI MSI

After sample preparation, ITO slides were locked in a Bruker MTP Slide Adapter II and immediately transferred into the vacuum system of an Ultraflex I MALDI TOF mass spectrometer equipped with a SmartBeam II Nd:YAG laser (Bruker Daltonik GmbH, Bremen, Germany). The laser spot size was set to 35 μm [14], while the laser fluence was optimized to obtain the best signal-to-noise (S/N) ratio and kept constant within a set of experiments. The MS data were acquired in the range from *m*/*z* 500 to 1000 by averaging 50 mass spectra at every single spot. In general, samples were rastered in the positive ion-mode first and subsequently, with an offset of approx. 50 μm, in the negative ion-mode at a lateral resolution of 110 × 110 μm. MSI raw data were processed utilizing the open source software mMass [24] as well as the MALDIquant (Version 1.16.2) [25] package and the Cardinal (Version 1.8.0) [26] package for the open-source R environment.

### Histological staining

After MSI data acquisition, the matrix was removed using ethanol. A standard protocol was then used to stain the aortic slices with hematoxylin and eosin (H&E) [27].

### LC-MS

High-resolution mass spectrometric data were obtained by performing LC-MS and LC-MS/MS analyses of methanolic extracts of aortic tissue slices in the positive as well as negative ion-mode (for details, see ESM) [28]. Lipid identification was performed by comparing accurate mass data with the LIPID MAPS structure database [29] and further confirmed by tandem MS measurements.

## Results and discussion

**Fig. 1:**
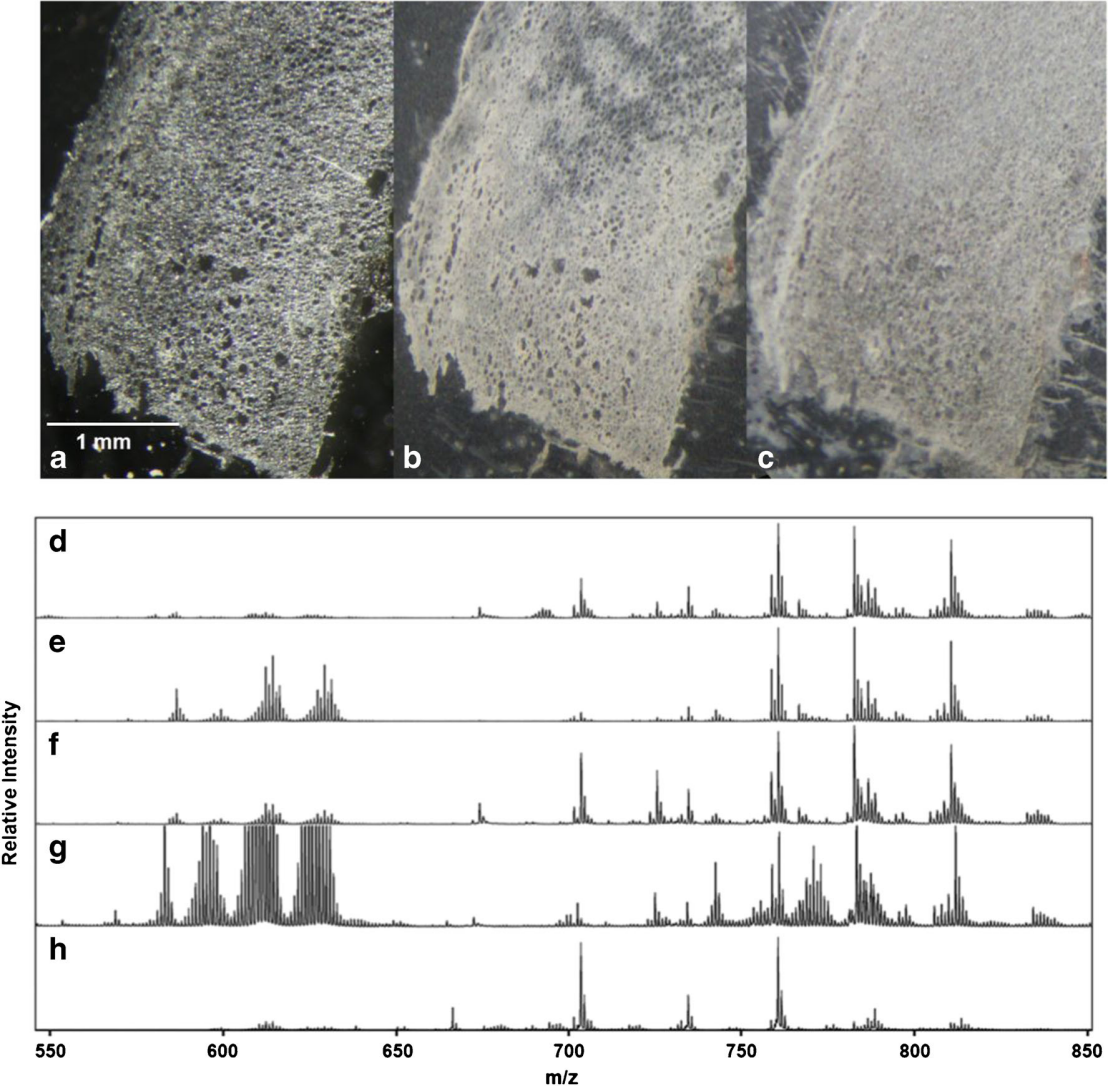
(**a**–**c**) Microscopic images of aortic tissue: (**a**) untreated tissue section, (**b**) tissue section after DAN matrix vapor deposition, and (**c**) after matrix recrystallization using toluene. (**d**–**h**) Effect of sample preparation on the signal intensities of lipid-related signals in the positive ion-mode. Average mass spectra in the range of *m*/*z* 550 to 850 were obtained by rastering whole tissue sections: (**d**) DAN matrix vapor deposition and recrystallization using toluene, (**e**) DAN matrix vapor deposition without recrystallization, (**f**) aortic tissue washed with NaCl solution prior to DAN matrix vapor deposition and recrystallization with toluene, (**g**) aortic tissue washed with NaCl solution prior to DAN matrix vapor deposition without recrystallization, (**h**) aortic tissue washed with NH_4_HCO_2_ solution prior to DAN matrix vapor deposition and recrystallization with toluene. For the negative mode, see the Electronic Supplementary Material

**Fig. 2:**
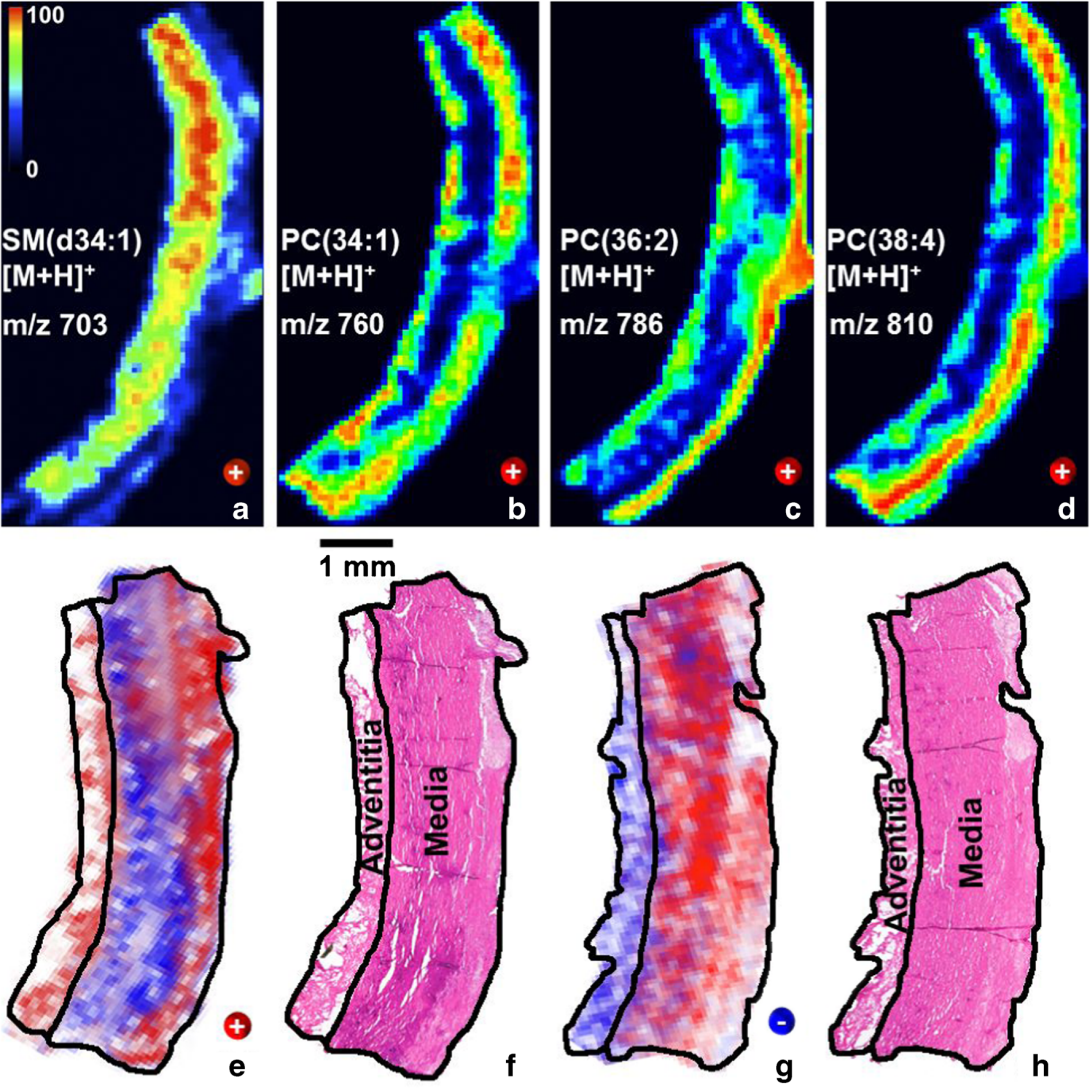
MALDI-TOF MSI of lipids in human aortic tissue. Cryosections (8 μm thick) were thaw-mounted on the ITO slides and coated with 1,5-DAN. After matrix recrystallization using toluene, the samples were rastered at a lateral resolution of 110 μm (laser spot size was set to 35 μm): (**a**–**d**) Positive mode ion images of SM(d34:1) at *m*/*z* 703 ([M+H]^+^), PC(34:1) at *m*/*z* 760 ([M+H]^+^), PC(36:2) at *m*/*z* 786 ([M+H]^+^), and PC(38:4) at *m*/*z* 810 ([M+H]^+^) are plotted using a color scale from black (0%) to red (100%); (**e**) positive mode ion images of PC(36:2) at *m*/*z* 786 ([M+H]^+^; red) superimposed by SM(d34:1) at *m*/*z* 703 ([M+H]^+^; blue) and the corresponding H&E stained tissue (**f**); (**g**) negative mode ion images of PC(34:4); at *m*/*z* 766 ([M−CH_3_]^−^; blue) superimposed by SM(d42:1) at *m*/*z* 799 ([M−CH_3_]^−^; red) and the corresponding H&E stained aortic tissue (**h**)

The thoracic aortic wall is composed of three different cell layers: the outer vascular wall (adventitia), the medial layer (media), and an inner monolayer (intima). Due to the small overall size of aortic cryosections of approx. 5–8 mm in length by 2 mm in width (see Figs. 1(a) and 2) and in order to exclude unwanted lateral analyte diffusion, we considered matrix vapor deposition/recrystallization [11, 18, 19] to be the method of choice for the production of highly homogenous matrix coatings for high-resolution MSI. While coatings obtained from widely used matrices such as 2,5-dihydroxybenzoic acid or α-cyano-4-hydroxycinnamic acid gave reasonable signal intensities for positive ions, these matrices turned out not to be feasible for the ionization of lipids in the negative mode [30].

According to literature, we obtained best results for imaging of lipids in aortic tissue in positive as well as negative ion-mode by using 1,5-DAN [30–32]. We used two different setups for matrix vapor deposition depending on the size of the solid support for the tissue (see ESM, Figs. S1 and S2). In any case, we avoided the cryosections to be exposed to open air at ambient temperature for more than a few seconds (for a photographic image of the matrix coating, see Fig. 1(b)). Additionally, during matrix deposition in vacuo, the samples were continuously cooled. Without matrix recrystallization, average spectra of positive ion-mode MALDI MSI showed 55 lipid-related signals in the range of *m*/*z* 650 to 850. We performed an automated peak picking with a signal-to-noise threshold > 6, followed by an automated deisotoping and manually excluded signals showing non-typical mass defects in a Kendrick mass defect plot [33–35]. However, in the range of *m*/*z* 570 to 650, a not negligible set of interfering signals was observed. Kendrick mass defect plots as well as further experiments revealed these clusters to originate from the DAN matrix (Fig. 1(e); Fig. S3 and Table S1 in the ESM). In order to suppress matrix-related signals and further enhance the sensitivity of the method, the effect of a matrix recrystallization was investigated. We tested different non-polar solvents such as chloroform, acetonitrile, and toluene. After matrix vapor deposition the sublimation apparatus was filled with a few milliliters of solvent, and the matrix coating was exposed to the vapor of the solvent at ambient pressure for about 10 to 30 s (for a photographic image of the recrystallized matrix coating on top of the aortic tissue, see Fig. 1(c)). The use of toluene as a solvent for recrystallization turned out to be most efficient. As depicted in Fig. 1(d), matrix cluster formation was found to be well suppressed while lipid-related signals stood out. A detailed comparison of the signal-to-noise ratios before and after recrystallization clearly indicated the advantages of a recrystallization step: under identical experimental conditions absolute signal intensities were found to be increased by 30–65% in the negative ion-mode, and from 5 to 23% in the positive ion-mode (see ESM, Fig. S7). Polarized light microscopic images of DAN coating showed that a recrystallization step transforms the amorphous coating into a microcrystalline surface structure. The crystal size of recrystallized DAN is estimated to be in the low micrometer range (see Fig. S5 in the ESM). Furthermore, we used pheophytin, a non-polar, colored, and toluene soluble chlorophyll derivative, to study the lateral analyte diffusion during recrystallization. Analyte diffusion turned out to be above 20 μm when the matrix was recrystallized for more than 30 s (see Fig. S6 of the ESM).

In order to control adduct formation in the positive ion-mode, the effect of tissue washing prior to matrix deposition was investigated. The use of physiological saline solution as a sodium donor increased the total number of lipid-related signals to 67, but did not reduce the abundance of protonated molecular ions. Moreover, matrix cluster formation turned out to be significantly enhanced, even after matrix recrystallization (Fig. 1(f, g)). On the other side, a tissue washing step using ammonium formate as a proton donor enhanced chemical noise throughout the spectrum and clearly reduced its signal-to-noise ratio. However, it successfully suppressed the formation of sodiated as well as potassiated molecular ions and turned out to be essential for signal assignment of the 48 lipid-related protonated molecular ions (Fig. 1(h) and ESM, Tables S1, S2, and S3).

Due to the lack of instrumental performance we obtained mass accuracies in the average mass spectra below 30 ppm. In order to avoid false assignments, we extracted two 8-μm tissue sections (adjacent to the imaged section) and performed LC-MS and LC-MS/MS experiments in positive and negative ion-mode. The obtained high-resolution data (see Electronic Supplementary Material) enabled the unambiguous determination of the elemental compositions of overall 55 different lipids and thus an internal recalibration of our TOF data. Moreover, our aortic tissue samples originate from a study done by Doppler et al. in 2017 where the authors published the identification and quantification of dozens of lipids using flow injection analysis (FIA) MS/MS [22]. Hence, based on three different sources of lipidomics data and considering standards of lipid identification and annotation [36], we were able to assign 34 lipid-related signals obtained from positive as well as negative ion-mode MALDI-TOF MSI (see ESM, Tables S2 and S3, Figs. S9A and S9B).

In conclusion, best results in terms of a significant enhancement of lipid-related signals (up to 65% in negative as well as up to 23% in positive ion-mode) for the detection of a maximum number of lipid-related signals in aortic tissue were obtained when untreated, thaw-mounted cryosections were coated with 1,5-DAN and toluene was used for recrystallization (see Fig. 2 and Figs. S3, S4, and S7 in the ESM). When an offset of a few micrometers is applied, the sample can consecutively be analyzed in the positive as well as negative ion-mode (ESM Fig. S9) [27, 30]. For lipid identification, a tissue washing using NH_4_HCO_2_ solution is recommended, which enforces the formation of protonated molecular ions.

As can be seen in Fig. 2, the tunica media layer of the aortic blood vessel is, according to H&E staining (2f and 2h), a uniform layer of cells. On the contrary, imaging mass spectrometry clearly reveals that on a molecular level, the media is by far not homogenous and features several so far unknown lateral distributions of different lipid species. Since lipids play essential roles in diverse tissues as constituents of cell membranes, act as regulators for various biological processes, and are associated with many diseases in which lipids show a modified distribution in tissue [37–40], our observations call for further investigations in order to clarify a possible physiological importance of lipid distribution in case of TAA.

In order to detect and identify even low abundant species using high-resolution MALDI MSI, a robust and fast sample preparation [36] featuring high S/N ratio as well as low chemical noise is essential. The presented method comprising an optional tissue washing followed by matrix vapor deposition/recrystallization provides a sample preparation within minutes and will easily be adapted for an application on different types of biologically relevant tissues.

## Electronic supplementary material

ESM 1 (PDF 2.36 mb)
