# Supplementary material for: Improved matrix coating for positive- and negative-ion-mode MALDI-TOF imaging of lipids in blood vessel tissues
Source: Anal Bioanal Chem. 2019 Apr 30;411(15):3221–7. doi: 10.1007/s00216-019-01826-x (PMC6542778; doi:10.1007/s00216-019-01826-x)
Supplement: Supplementary file 1 — (PDF 2.36 mb) [file 216_2019_1826_MOESM1_ESM.pdf]

Analytical and Bioanalytical Chemistry

Electronic Supplementary Material

Improved matrix coating for positive- and negative-ion-mode MALDI-TOF imaging of lipids in blood vessel tissues

Christina Meisenbichler, Christian Doppler, David Bernhard, Thomas Müller

Matrix vapor deposition and recrystallization on small substrates

Matrix deposition on small sized stainless steel or ITO targets was carried out using a commercially available sublimation apparatus (Figure S1). The apparatus was filled with about 200 mg of 1,5-DAN, while the stainless steel or ITO target with an aortic tissue section was fixed at the cooling finger using adhesive tape. The sublimation apparatus was coupled to a rough pump, a liquid chiller and was placed in an oil bath. Sublimation was performed at an oil bath temperature of 144 °C and a rough vacuum <2x10^{-3} mbar for 2.5 minutes. The sublimation apparatus was then opened, the matrix removed and the flask was filled with 2 mL of e.g. acetonitrile, chloroform or toluene. The cooling finger was inserted into the flask with the boiling solvent to expose the matrix coating to the vapor for about 10 seconds. The metal slide was fixed on a MTP ground steel target using an UV curing glue and directly transferred into the vacuum system of the mass spectrometer.

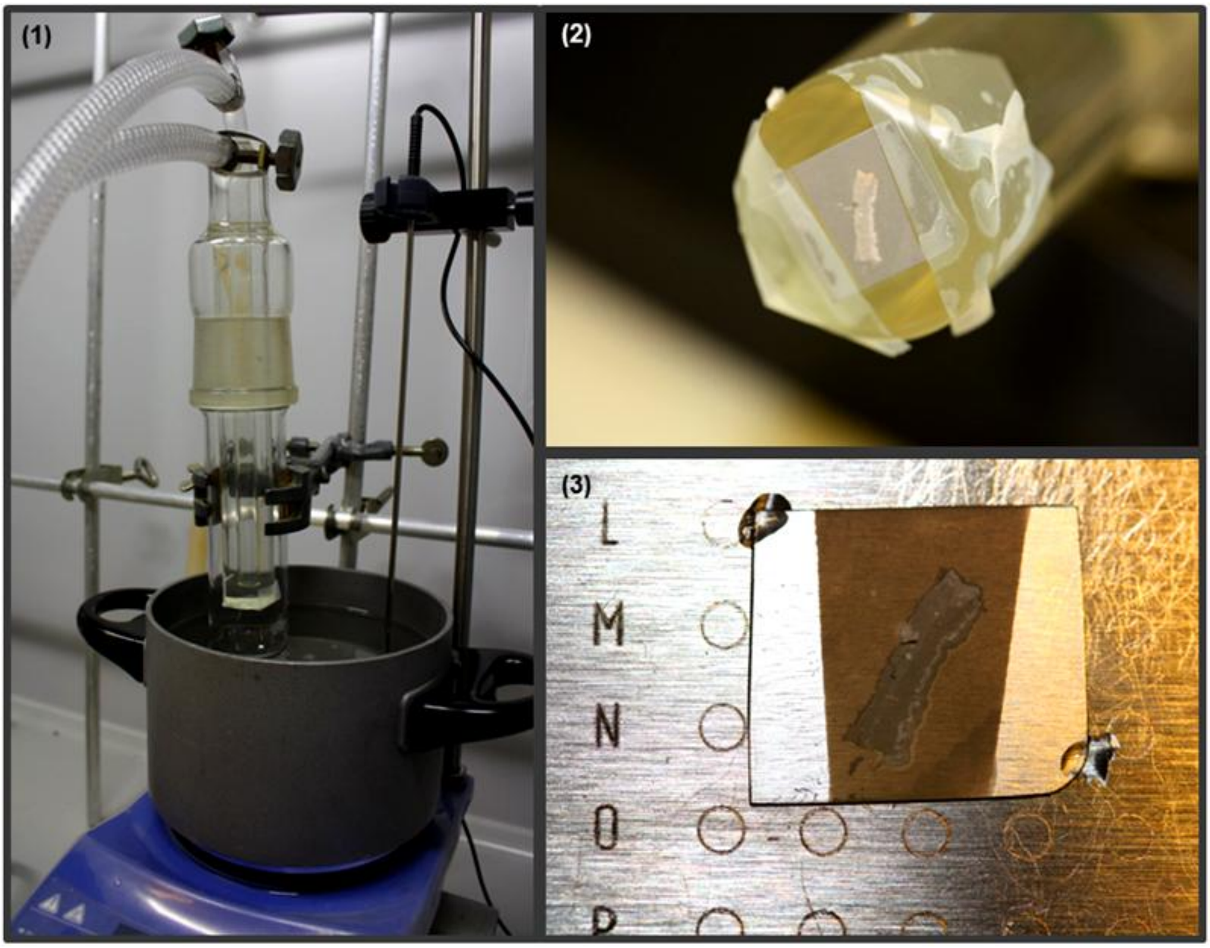

Fig. S1 Experimental setup for matrix vapor deposition/recrystallization on small sized stainless steel or ITO glass slides. The commercially available sublimation apparatus on top of an oil bath, the attached rough pump and the chiller are not shown (1); small sized stainless-steel target with an aortic tissue section fixed at the cooling finger using an adhesive tape (2); small sized stainless-steel target fixed on the stainless steel MALDI target using an UV curing glue (3)

Matrix vapor deposition and recrystallization on ITO slides

Matrix deposition on ITO glass slides was carried out using in-house built sublimation apparatus (Figure S2). A petri dish on top of a heating plate was filled with about 200 mg of 1,5-DAN, while the ITO slide with an aortic tissue section was fixed on the cooling plate using adhesive tape. The sublimation apparatus was closed and coupled to a rough pump and a liquid chiller (15°C). Sublimation was performed within 5 minutes at a rising heating plate temperature between 123 - 136 °C and a rough vacuum $<2x10^{-3}$ mbar. According to a method described by Yang et al. [1], the amount of deposited matrix was determined to be 0.111 ± 0.004 $mgxcm^{-2}$. The sublimation apparatus was then opened, the petri dish with the matrix removed and the hot plate quickly cooled down to about 20°C above the boiling point of the solvent for recrystallization. Another petri dish filled with 5 mL of e.g. acetonitrile, chloroform or toluene was placed on the hot plate and the sublimation apparatus was closed for about 30 seconds to expose the matrix coating to the vapor of the boiling solvent. The ITO slide was fixed in a Bruker MTP Slide Adapter II and directly transferred into the vacuum system of the mass spectrometer.

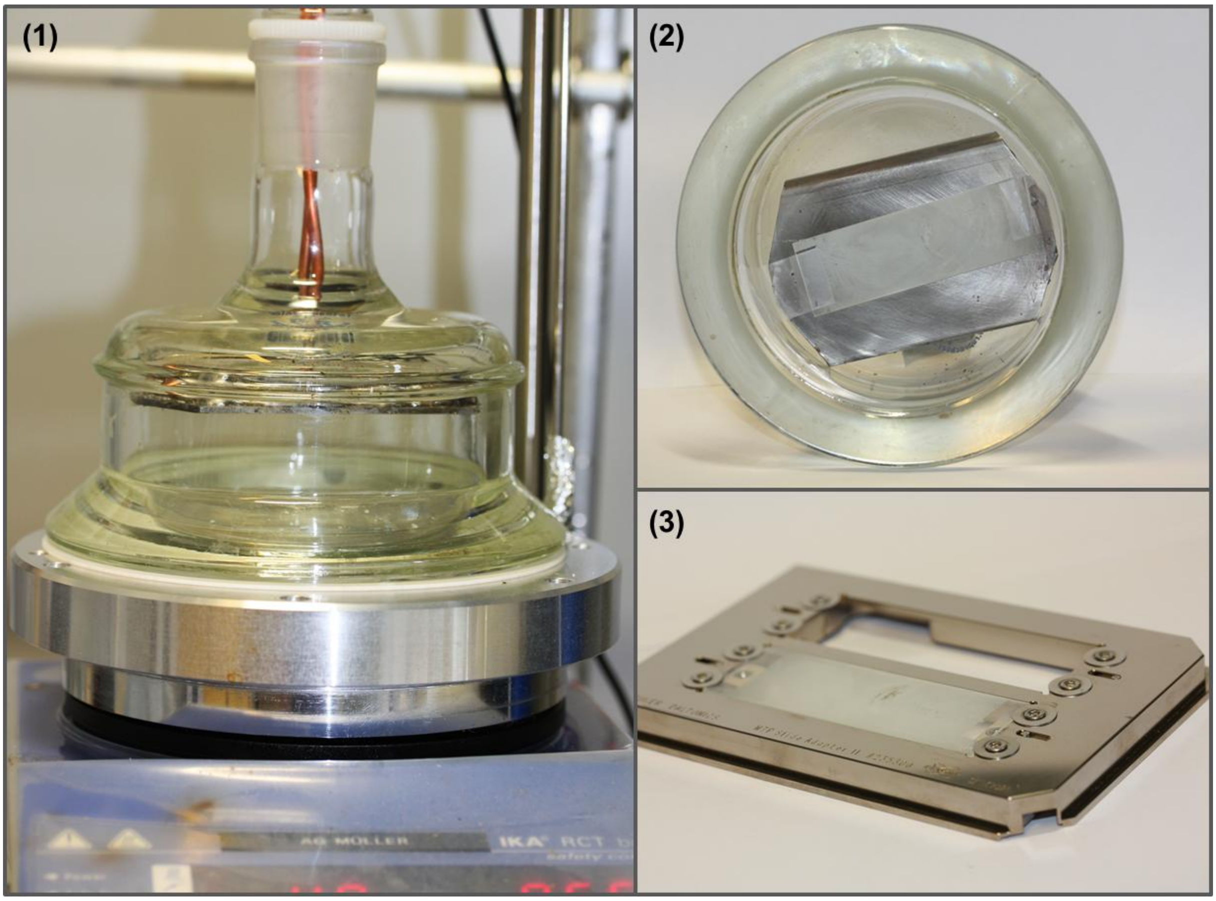

Fig. S2 Experimental setup for matrix vapor deposition/recrystallization on standard ITO glass slides. The in-house built sublimation apparatus on top of a commercially available hot plate, the attached rough pump and the chiller are not shown (1); ITO glass slide with an aortic tissue section fixed at the cooling plate using adhesive tape (2); ITO glass slide inside the Bruker MTP Slide Adapter II (3)

Optimization of sample preparation for MALDI-MSI

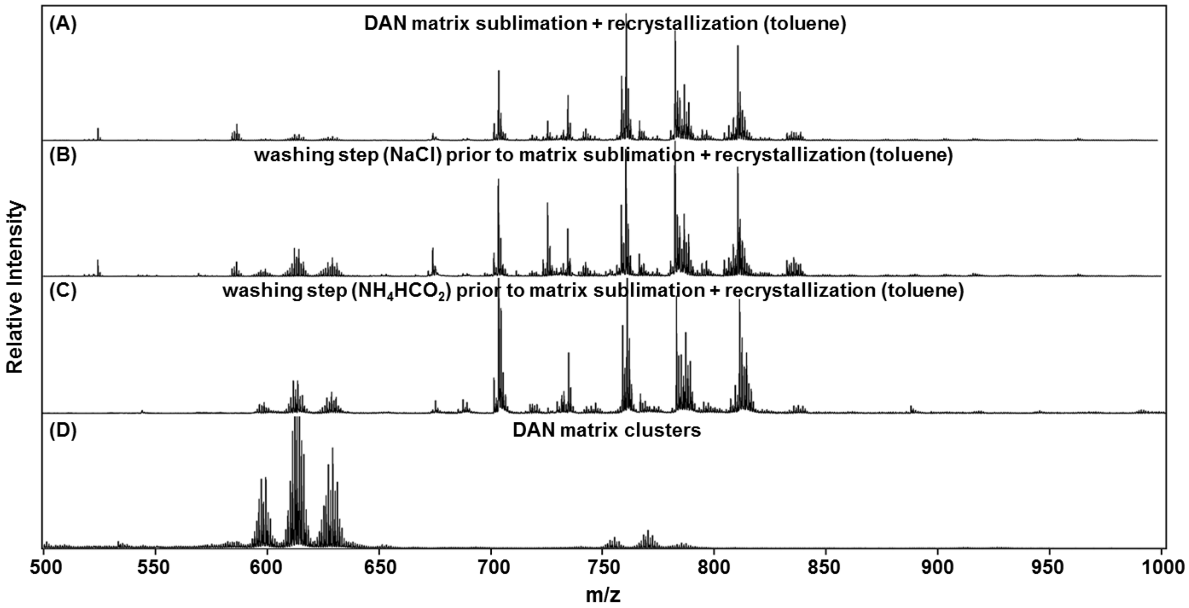

Fig. S3 Effect of sample preparation on the signal intensities of matrix and lipid related signals in the positive ion-mode: Average mass spectra obtained from (A) aortic tissue, DAN matrix vapor deposition and recrystallization using toluene; (B) aortic tissue washed with NaCl solution prior to DAN matrix vapor deposition and recrystallization with toluene; (C) aortic tissue washed with NH_4HCO_2 solution prior to DAN matrix vapor deposition and recrystallization with toluene; (D) an empty ITO slide, DAN matrix vapor deposition

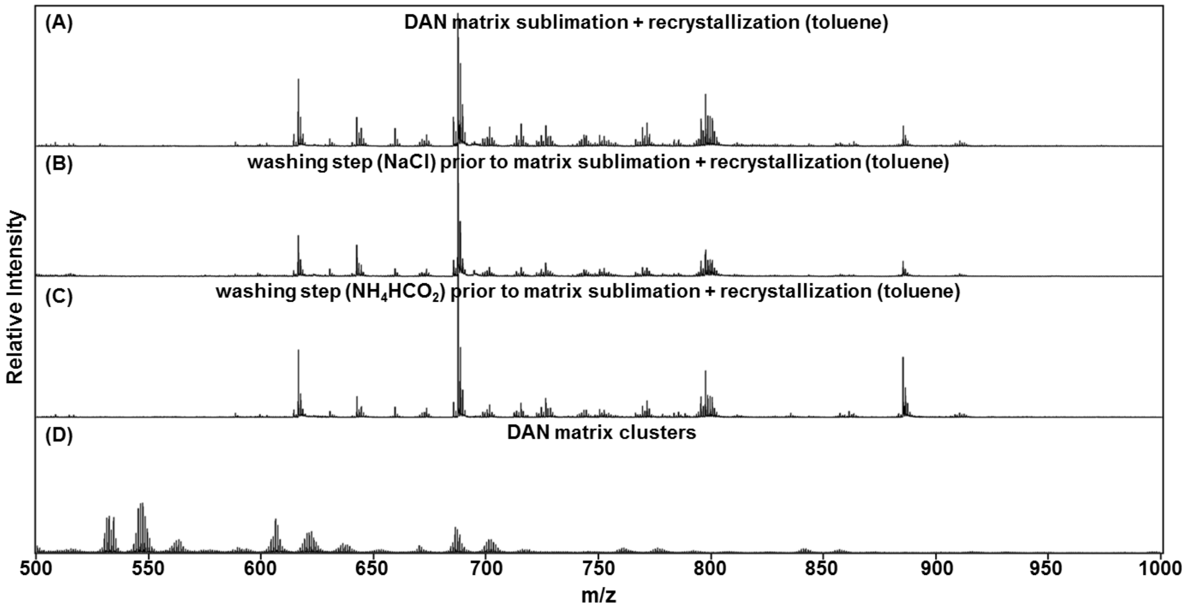

Fig. S4 Effect of sample preparation on the signal intensities of matrix and lipid related signals in the negative ion-mode: Average mass spectra obtained from (A) aortic tissue, DAN matrix vapor deposition and recrystallization using toluene; (B) aortic tissue washed with NaCl solution prior to DAN matrix vapor deposition and recrystallization with toluene; (C) aortic tissue washed with NH_4HCO_2 solution prior to DAN matrix vapor deposition and recrystallization with toluene; (D) an empty ITO slide, DAN matrix vapor deposition

Table S1 Validation of different sample preparation procedures. Open source mass spectrometry tool mMass[2] was used to process average spectra (whole sample). After automated peak picking (with a signal to noise threshold >6) and deisotoping a Kendrick mass defect plot[3] was utilized to determine the number of lipid related signals as well as to exclude signals, which are obviously not related to lipid compounds

Sample preparation	Lipid related signals from m/z 650 to 850 (positive ion-mode)
DAN vapor deposition	55
DAN vapor deposition/recrystallization toluene	57
NaCl wash, DAN vapor deposition/recrystallization toluene	67
NH_4HCO_2 wash, DAN vapor deposition/recrystallization toluene	48

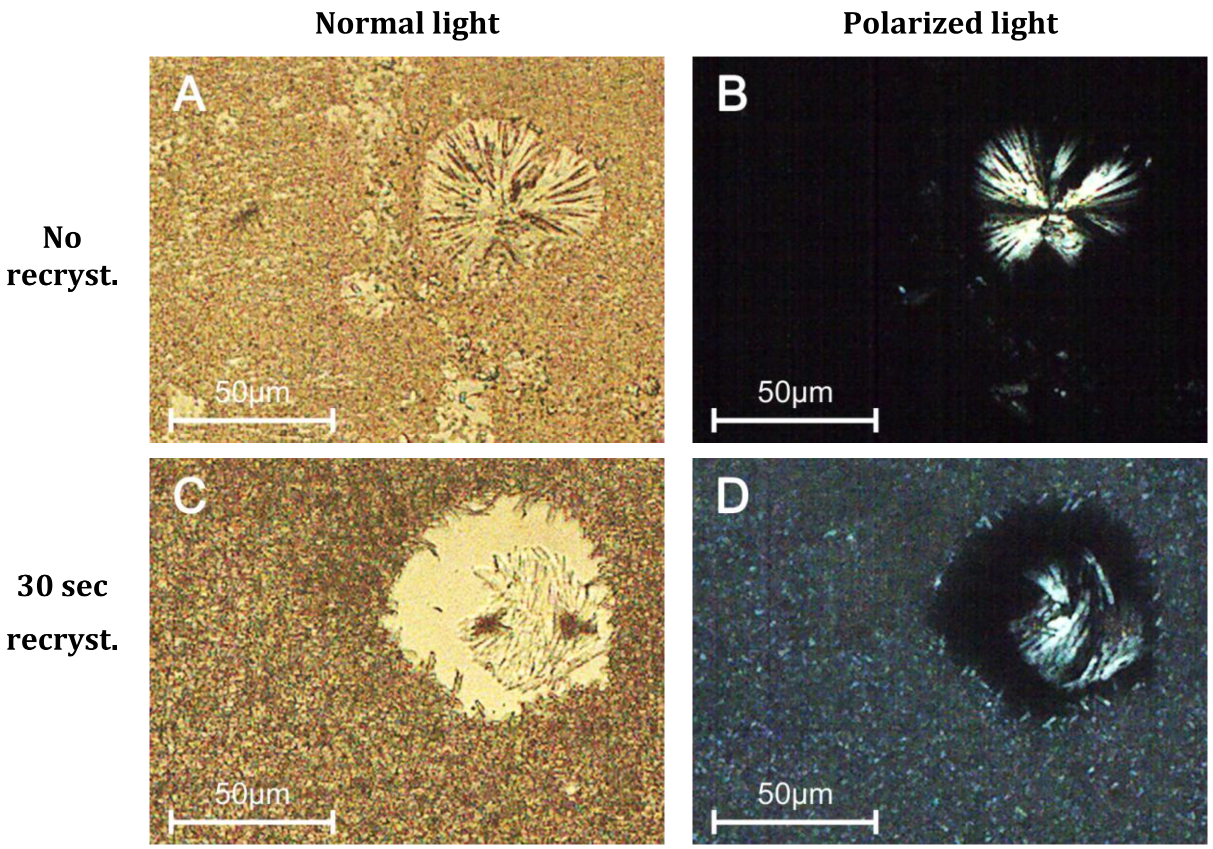

Fig. S5 Effect of matrix recrystallization on the DAN matrix coating. Microscopic images of the matrix coating deposited on glass slides were taken using a polarized light microscope (Olympus BX40 with a 40x/0.65na objective): Without recrystallization (A and B) some scattered, crystalline spherical structures (40-50 µm diameter) were observed while most of coating appeared to be amorphous (black area). After 30 sec of recrystallization using toluene (C and D) crystalline matrix was found throughout the sample surface. The crystal size is estimated to be below 10 µm

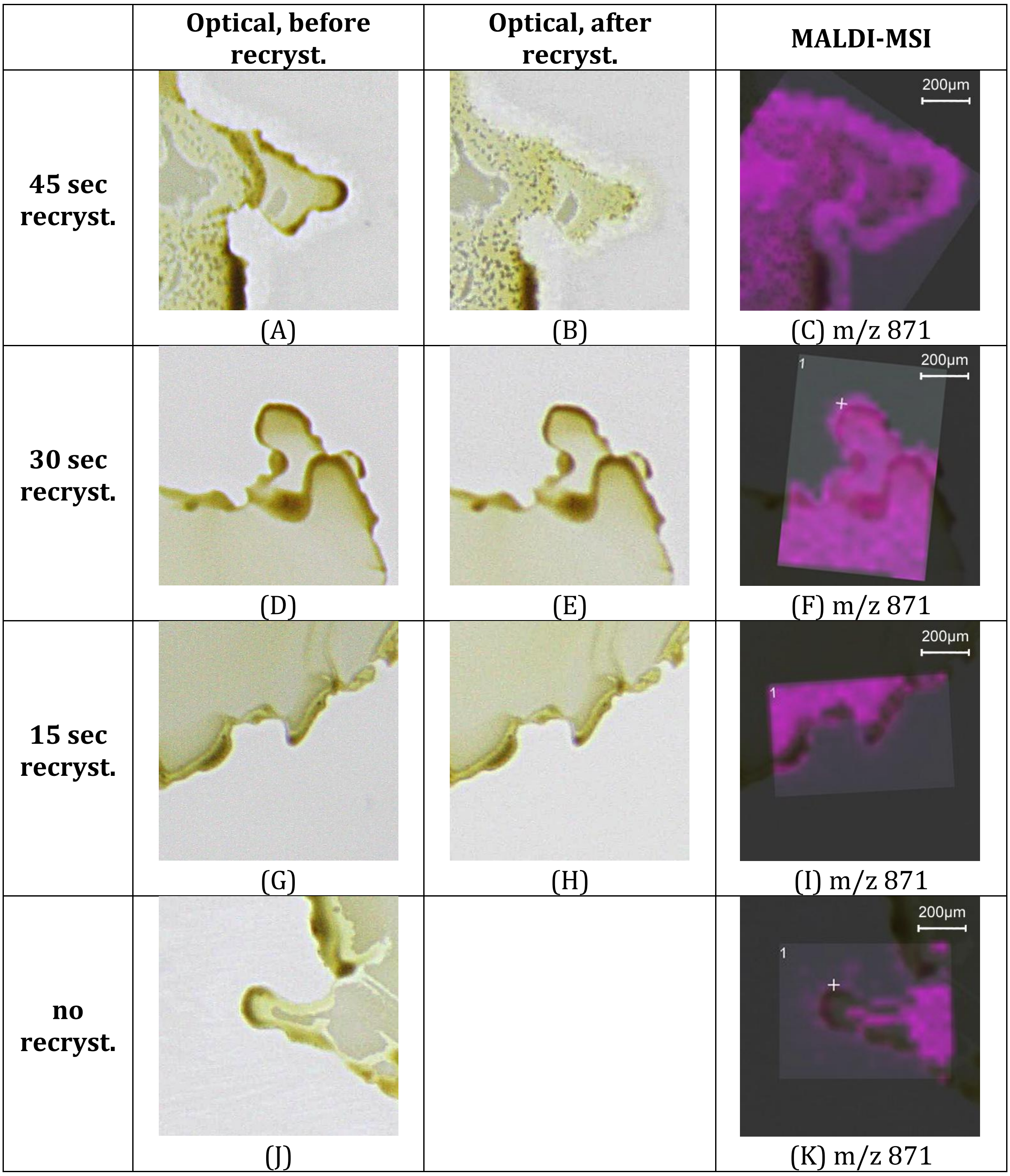

Fig. S6 Effect of DAN recrystallization using toluene on the lateral diffusion of a non-polar analyte. Pheophytin "a", a colored natural product, which is perfectly soluble in toluene, was dissolved in toluene and spotted onto four different ITO glass slides. After 5 minutes of DAN matrix vapor deposition microscopic images of selected spots were taken (panels A, D, G and J). After 45 sec, 30 sec and 15 sec of matrix recrystallization using toluene further microscopic images of the same spots were taken (panels B, E and H) and MALDI MSI in the positive ion-mode (as described in Materials and Methods part of the main text) was performed at a lateral resolution of 35 µm: (C) 45 sec recrystallization using toluene resulted in an unacceptable lateral analyte diffusion of approx. 100 µm; (F) optimum conditions were found at 30 sec recrystallization resulting in an acceptable lateral diffusion below 20 µm and high signal-to-noise ratios throughout the sample; (I and K) very short or no recrystallization showed no lateral analyte diffusion on the one side, but caused signal suppression due to lack of orthogonal analyte integration at the peripheral zones of the pheophytin spots

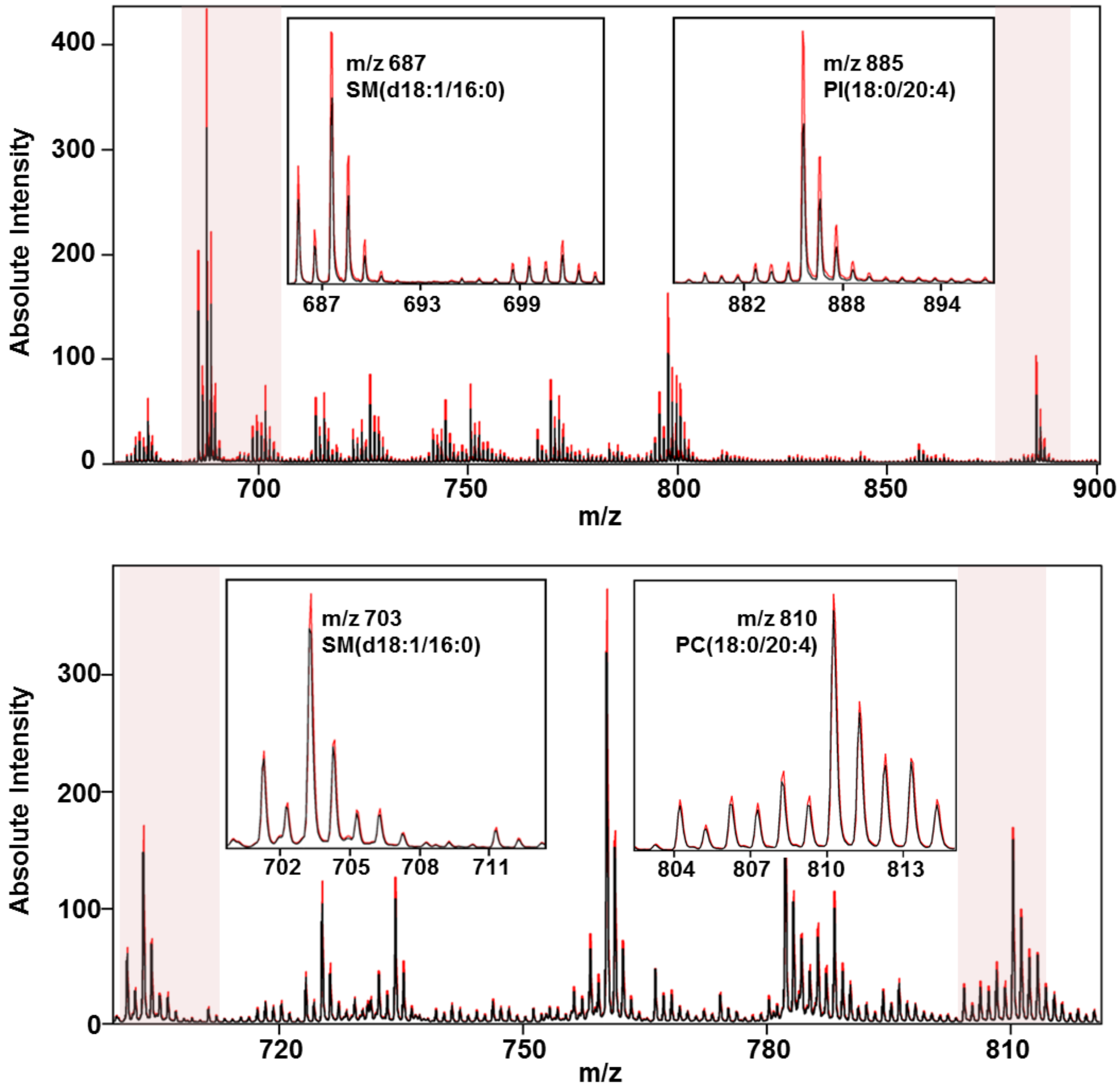

Fig. S7 Effect of recrystallization on the signal-to-noise ratio. For a better comparison two adjacent cryosections of human aortic tissue (8 µm thick) were thaw-mounted on the same ITO slide and coated with 1,5-DAN. While one section was covered with a piece of paper, the coating of the second section was recrystallized using toluene. The samples were subjected to MALDI MSI analysis and rastered in positive as well as negative ion-mode under equal conditions (identical laser fluence; lateral resolution was set to 70 µm; 20 shot per spot; random walk after 5 consecutive shots). Average spectra were obtained from about ¼ of each aortic section comprising all layers of the tissue (black line = without recrystallization; red line = with recrystallization). In the negative ion-mode (top) absolute signal intensities of lipid related signals were found to be increased by 30-65%, in the positive ion-mode (bottom) absolute signal intensities of lipid related signals were found to be increased by 5-23%

Processing MSI data and data visualization

MSI raw data were first exported to the common Analyze 7.5 format using FlexImaging (Bruker Daltonics) and then processed utilizing the open-source software mMass[2] as well as MALDIquant (Version 1.16.2)[4] and Cardinal (Version 1.8.0)[5] packages for the R environment. An in-house R script was developed to generate average spectra, preprocess and visualize the MSI data set.

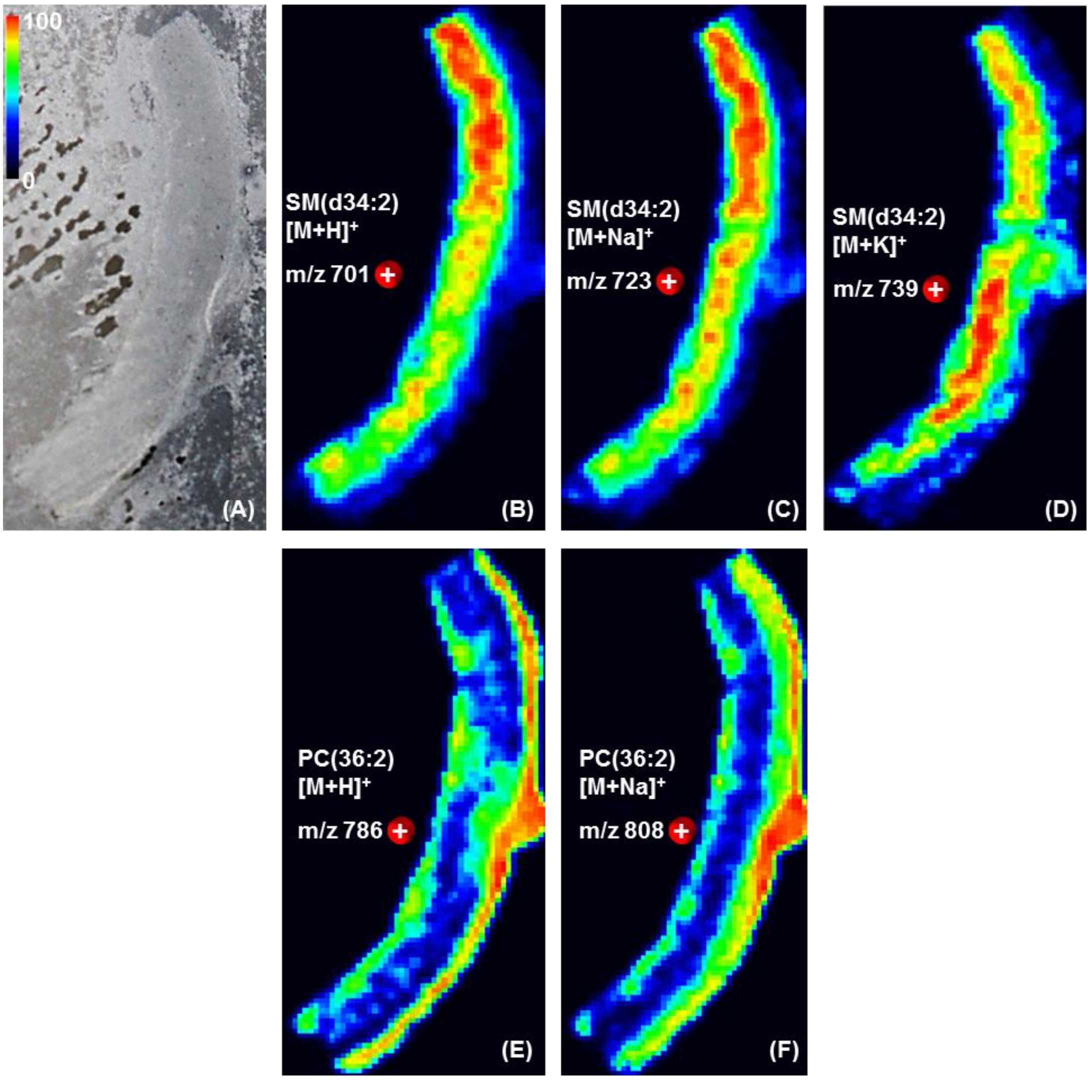

Fig. S8 MALDI-TOF MSI of lipids in human aortic tissue. Cryosections (8 µm thick) were thaw-mounted on the ITO slides and coated with 1,5-DAN. After matrix recrystallization using toluene the samples were rastered at a lateral resolution of 110 µm (laser spot size was set to 35 µm): (A) Photographic image of the matrix coated aortic tissue; (B-D) positive mode ion images of the protonated, sodiated and potassiated molecular ions of SM(d34:2) at m/z 701 ($[M+H]^+$), m/z 723 ($[M+Na]^+$), m/z 739 ($[M+K]^+$); (E,F) positive mode ion images of the protonated and sodiated molecular ions of PC(36:2) at m/z 786 ($[M+H]^+$) and m/z 808 ($[M+Na]^+$). All images are plotted using a color scale from black (0%) to red (100%)

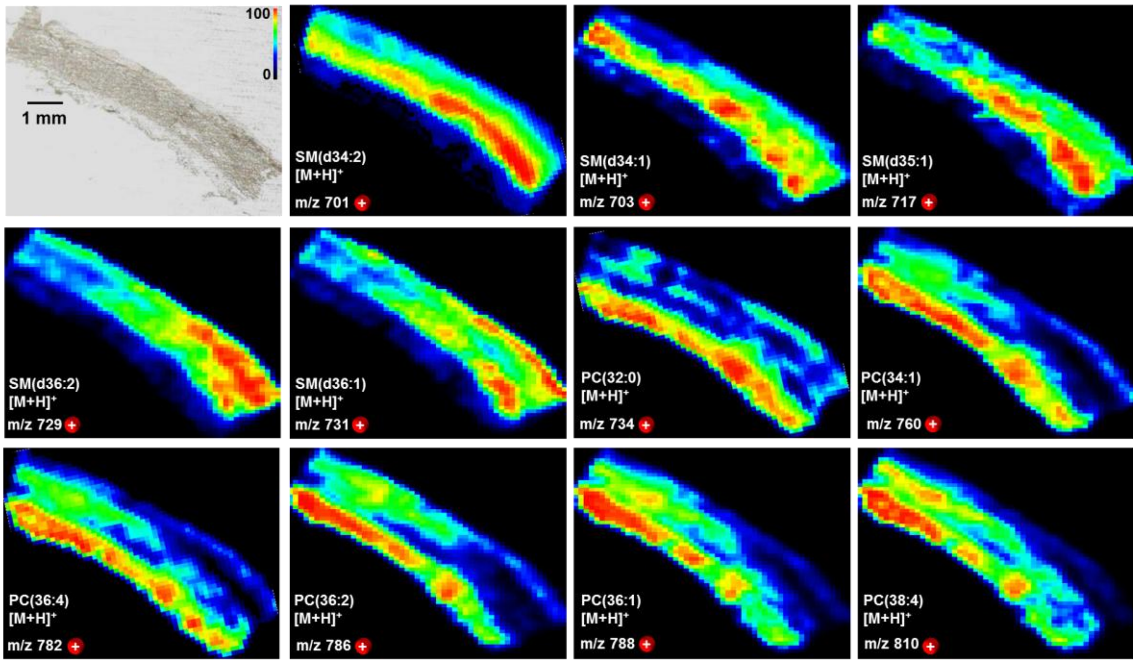

Fig. S9A Positive ion-mode MALDI-TOF MSI of lipids in human aortic tissue. A cryosection (8 µm thick) was thaw-mounted on a stainless steel slide and coated with 1,5-DAN. After matrix recrystallization using toluene the sample was rastered in the positive and then, with an offset of approx. 50µm, in the negative mode at a lateral resolution of 110 µm (laser spot size was set to 35 µm). From top left to bottom right: Photographic image of the matrix coated aortic tissue and all positive mode ion images of lipids listed in Table S2. All images are plotted using a color scale from black (0%) to red (100%). Corresponding negative ion-mode images are depicted in Fig. S9B

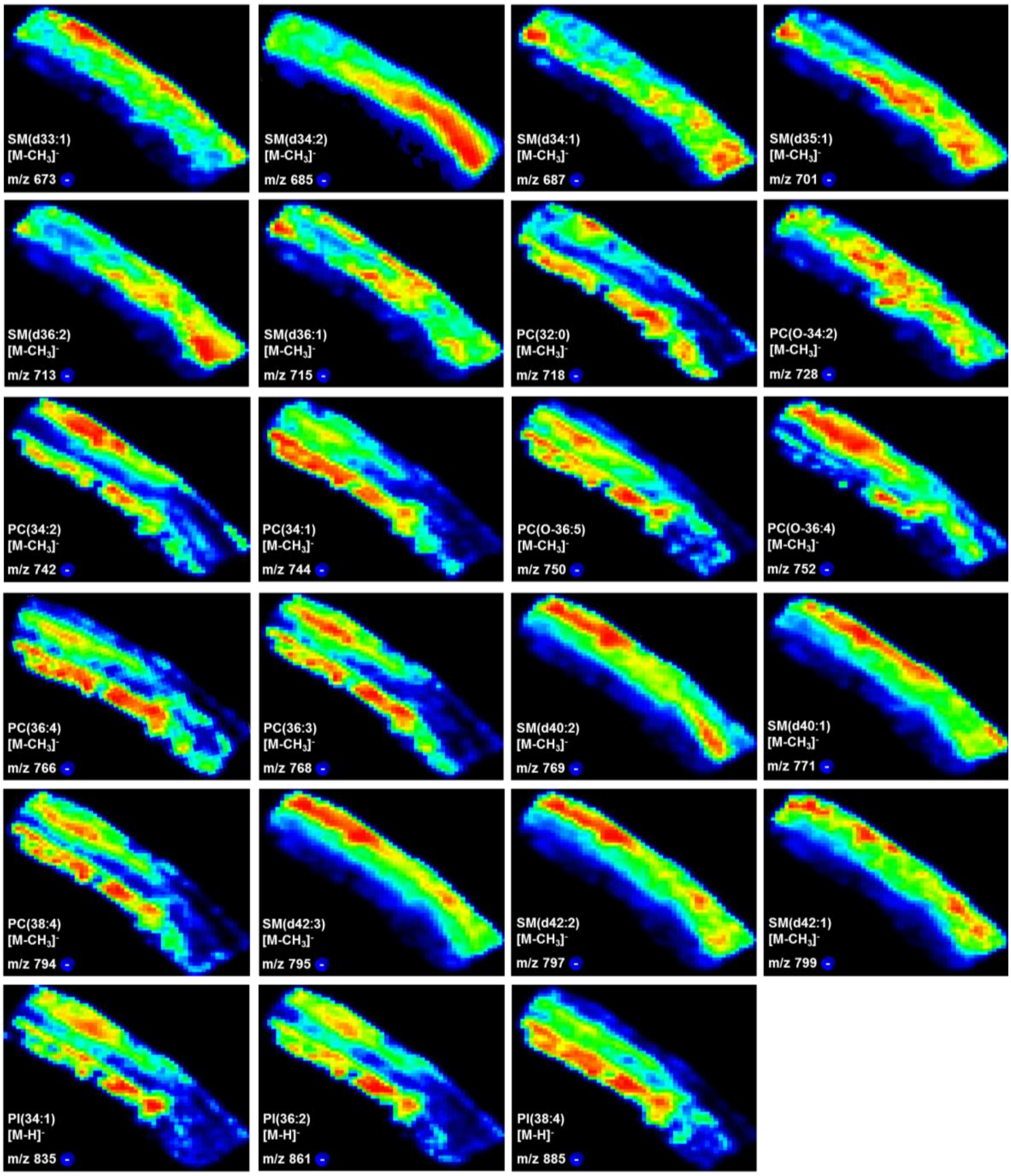

Fig. S9B Consecutive negative ion-mode MALDI-TOF MSI of lipids in human aortic tissue. A cryosection (8 µm thick) was thaw-mounted on a stainless steel slide and coated with 1,5-DAN. After matrix recrystallization using toluene the sample was rastered in the positive and then, with an offset of approx. 50µm, in the negative mode at a lateral resolution of 110 µm (laser spot size was set to 35 µm). From top left to bottom right: All negative mode ion images of lipids listed in Table S3. All images are plotted using a color scale from black (0%) to red (100%). Corresponding positive ion-mode images are depicted in Fig. S9A

MALDI MSI: Assignment of lipids

Software mMass[2] was used to process average spectra obtained from an aortic tissue section washed with NH_4HCO_2 solution prior to DAN matrix sublimation and recrystallization using toluene. After automated peak picking (signal-to-noise threshold >6) and deisotoping a Kendrick mass defect plot[3, 6, 7] was utilized to identify lipid related signals. The signal assignment is based on a study from Doppler et al.[8], who published a quantification of metabolites in the same aortic tissue samples using AbsoluteIDQ1 p150 kit (BIOCRATES Life Sciences AG, Innsbruck, Austria) and was further validated by an LC-MS/MS analysis (see below).

Table S2 Positive ion-mode MALDI MSI: Protonated molecular ions ($[M+H]^+$) of 11 identified lipids in human aortic tissue. The assignment is based on [8] as well as on LC-MS/MS analyses described below. Lipid related signals, which were not unambiguously assigned due to lack of mass spectrometric resolution, are not listed

m/z	Assignment due to [8]	Assignment due to LC-MS/MS	Formula $[M+H]^+$	$m/z_{(calc)}$	Δ(ppm)
701.56	SM(d18:1/16:1)	SM(d18:1/16:1)	$C_{39}H_{78}N_2O_6P$	701.5592	-2.7
703.57	SM(d18:1/16:0)	SM(d18:1/16:0)	$C_{39}H_{80}N_2O_6P$	703.5749	-8.8
717.57	SM(d18:1/17:0)	SM(d18:1/17:0)	$C_{40}H_{82}N_2O_6P$	717.5905	-28.2
729.58	SM(d18:1/18:1)	SM(d18:1/18:1)	$C_{41}H_{82}N_2O_6P$	729.5905	-15.6
731.59	SM(d18:1/18:0)	SM(d18:1/18:0)	$C_{41}H_{84}N_2O_6P$	731.6062	-23.5
734.59	PC(32:0)	PC(16:0/16:0)	$C_{40}H_{81}NO_8P$	734.5694	23.9
760.60	PC(34:1)	PC(16:0/18:1)	$C_{42}H_{83}NO_8P$	760.5851	13.6
782.57	PC(36:4)	PC(16:0/20:4)	$C_{44}H_{81}NO_8P$	782.5694	0.1
786.62	PC(36:2)	PC(18:0/18:2)	$C_{44}H_{85}NO_8P$	786.6007	28.0
788.62	PC(36:1)	PC(36:1)	$C_{44}H_{87}NO_8P$	788.6164	7.3
810.59	PC(38:4)	PC(18:0/20:4)	$C_{46}H_{85}NO_8P$	810.6007	-10.7

Table S3 Negative ion-mode MALDI MSI: Fragment ions ($[M-CH_3]^-$) as well as deprotonated molecular ions ($[M-H]^-$; marked with "#") of 23 lipids identified in human aortic tissue. The assignment is based on [8] as well as on LC-MS/MS analyses described below. Lipid related signals, which were not unambiguously assigned due to lack of mass spectrometric resolution, are not listed

m/z	Assignment due to [8]	Assignment due to LC-MS/MS	Formula	$m/z_{(calc)}$	Δ(ppm)
673.53	SM(d18:1/15:0)	SM(d18:1/15:0)	$C_{37}H_{74}N_2O_6P$	673.5290	-4.8
685.53	SM(d18:1/16:1)	SM(d18:1/16:1)	$C_{38}H_{74}N_2O_6P$	685.5290	1.0
687.54	SM(d18:1/16:0)	SM(d18:1/16:0)	$C_{38}H_{76}N_2O_6P$	687.5447	-0.9
701.54	SM(d18:1/17:0)	SM(d18:1/17:0)	$C_{39}H_{78}N_2O_6P$	701.5603	-23.6
713.55	SM(d18:1/18:1)	SM(d18:1/18:1)	$C_{40}H_{78}N_2O_6P$	713.5603	-11.4
715.57	SM(d18:1/18:0)	SM(d18:1/18:0)	$C_{40}H_{80}N_2O_6P$	715.5760	-10.1
718.53	PC(32:0)	PC(16:0/16:0)	$C_{39}H_{77}NO_8P$	718.5392	-10.3
728.57	PC(O-34:2)		$C_{41}H_{79}NO_7P$	728.5600	11.8
742.55	PC(34:2)	PC(16:0/18:2)	$C_{41}H_{77}NO_8P$	742.5392	8.4
744.55	PC(34:1)	PC(16:0/18:1)	$C_{41}H_{79}NO_8P$	744.5549	-0.6
750.53	PC(O-36:5)		$C_{43}H_{77}NO_7P$	750.5443	-14.0
752.56	PC(O-36:4)		$C_{43}H_{79}NO_7P$	752.5600	6.0
766.52	PC(36:4)	PC(16:0/20:4)	$C_{43}H_{77}NO_8P$	766.5392	-26.5
768.54	PC(36:3)		$C_{43}H_{79}NO_8P$	768.5549	-14.0
769.62	SM(d18:1/22:1)		$C_{44}H_{86}N_2O_6P$	769.6229	-8.4
771.63	SM(d18:1/22:0)	SM(d18:1/22:0)	$C_{44}H_{88}N_2O_6P$	771.6386	-8.6
794.57	PC(38:4)	PC(18:0/20:4)	$C_{45}H_{81}NO_8P$	794.5705	1.2
795.64	SM(d18:1/24:2)		$C_{46}H_{88}N_2O_6P$	795.6380	6.0
797.66	SM(d18:1/24:1)	SM(d18:1/24:1)	$C_{46}H_{90}N_2O_6P$	797.6542	11.7
799.68	SM(d18:1/24:0)	SM(d18:1/24:0)	$C_{46}H_{92}N_2O_6P$	799.6699	8.5
835.53		PI(34:1)	$C_{43}H_{80}O_{13}P^{\#}$	835.5342	-1,4
861.56		PI(36:2)	$C_{45}H_{82}O_{13}P^{\#}$	861.5499	8.7
885.56		PI(18:0/20:4)	$C_{47}H_{82}O_{13}P^{\#}$	885.5499	9.5

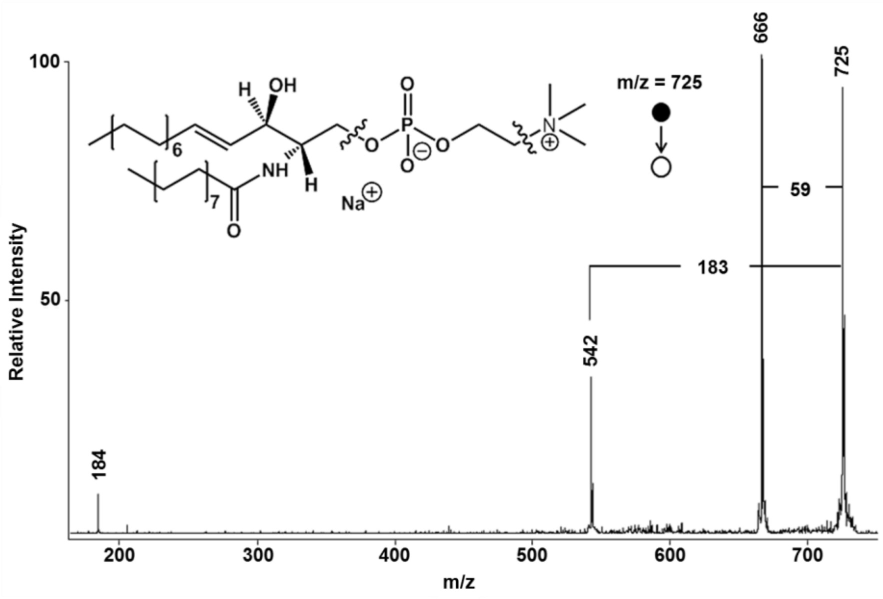

Fig. S10 Example of lipid class identification using on-tissue MALDI-TOF PSD. The lipid class of the compound related to the signal at m/z 725 was identified by performing Post Source Decay (PSD) experiments on the isolated ions[9] (isolation window was 10 Da). PSD fragmentation is dominated by a loss of 59 Da, which correspond to a cleavage at the quaternary ammonium residue, and a loss of 183 Da, which correspond to a cleavage of a phosphor ester bond. Based on further LC-MS/MS data (see below) the signal at m/z 725 was assigned to the sodiated molecular ion of the sphingomyelin SM(d34:1). Due to data from Doppler et al.[8] SM(d34:1) is SM(d18:1/16:0) as depicted

Identification of Lipids in human aortic tissue using LC-MS/MS

Lipid extraction of human aortic tissue slices

Two human aortic tissue slices (8 µm each) were detached from glass slides using a small spatula and transferred into an Eppendorf microcentrifuge tube. 100 µL of MeOH were added and the suspension was sonicated for 5 minutes and vortexed for additional 20 sec. The suspension was then centrifuged at 13400 rpm for 10 min. The lipid containing organic supernatant was transferred into a fresh tube and stored at -20°C until use.

LC-MS and LC-MS/MS

For LC-MS and LC-MS/MS analyses of the extracted lipids a Shimadzu HPLC-system coupled to a ThermoScientific LTQ Orbitrap XL mass spectrometer (positive ion-mode) and a ThermoScientific Q Exactive Hybrid Quadrupole-Orbitrap mass spectrometer (negative ion-mode) was used. Slightly different backpressures at the two ESI sources caused a 0.5 min shift of the retention times, which were determined from the extracted-ion chromatograms depicted in Fig. S11. The HPLC system was equipped with an LC-20AD pump, a DGU-20A5 online degasser unit, a SPD-M20A diode array detector, a Rheodyne injection valve with a 20 µL injection loop, and an Eclipse XDB-C18 column (ODS 3.5 µm 100 x 3.0 mm I.D.). At a flow rate of 0.5 mLxmin^{-1} a gradient adapted from Sato et al.[10] from 35% of solvent A (60:27:13 H_2O/ACN/MeOH, 10 mM ammonium formate, 0.1% FA) to 100% of solvent B (80:20 IPA/MeOH, 10 mM ammonium formate, 0.1% FA) was used (A/B (v/v)): 0-1 min: 35/65; 1-2 min 35/65 to 27/73; 2-4 min 27/73 to 23/77; 4-7 min 23/77 to 20/80; 7-9 min 20/80 to 16.5/83.5; 9-11 min 16.5/83.5 to 14/86; 11-15 min 14/86 to 0/100; 15-25 min 0/100. Data were collected and processed using Shimadzu LC Solution software (version 1.24 SP1). The mass spectrometers were equipped with ESI sources and were set up for data dependent acquisition of MS/MS spectra. Data were collected with Xcalibur 2.2 software (Thermo Fisher Scientific Inc).

Table S4 High-resolution LC-ESI-MS and LC-ESI-MS/MS in positive ion-mode: Protonated molecular ions ($[M+H]^+$), sodium adducts ($[M+Na]^+$), potassium adducts ($[M+K]^+$) as well as ammonium adducts ($[M+NH_4]^+$) of 35 unambiguously identified lipids in methanolic extracts of human aortic tissue slices

m/z	Annotation	Formula	Ion	$m/z_{(calc)}$	t_R
711.5411	SM(d18:1/15:0)	$C_{38}H_{77}N_2O_6PNa$	$[M+Na]^+$	711.5411	7.8
723.5410	SM(d18:1/16:1)	$C_{39}H_{77}N_2O_6PNa$	$[M+Na]^+$	723.5411	7.4
725.5569	SM(d18:1/16:0)	$C_{39}H_{79}N_2O_6PNa$	$[M+Na]^+$	725.5568	8.6
734.5685	PC(16:0/16:0)	$C_{40}H_{81}NO_8P$	$[M+H]^+$	734.5694	10.6
739.5720	SM(d18:1/17:0)	$C_{40}H_{81}N_2O_6PNa$	$[M+Na]^+$	739.5724	9.4
741.5317	SM(d18:1/16:0)	$C_{39}H_{79}N_2O_6PK$	$[M+K]^+$	741.5307	8.6
751.5730	SM(d18:1/18:1)	$C_{41}H_{81}N_2O_6PNa$	$[M+Na]^+$	751.5724	9.0
753.5878	SM(d18:1/18:0)	$C_{41}H_{83}N_2O_6PNa$	$[M+Na]^+$	753.5881	10.3
756.5514	PC(16:0/16:0)	$C_{40}H_{80}NO_8PNa$	$[M+Na]^+$	756.5514	10.6
760.5850	PC(16:0/18:1)	$C_{42}H_{83}NO_8P$	$[M+H]^+$	760.5851	10.8

762.6004	PC(16:0/18:0)	$C_{42}H_{85}NO_8P$	$[M+H]^+$	762.6007	12.4
768.7079	TG(44:0)	$C_{47}H_{94}NO_6$	$[M+NH_4]^+$	768.7076	19.1
781.6192	SM(d18:1/20:0)	$C_{43}H_{87}N_2O_6PNa$	$[M+Na]^+$	781.6194	12.1
782.5674	PC(16:0/18:1)	$C_{42}H_{82}NO_8PNa$	$[M+Na]^+$	782.5670	10.8
785.6060	TG(44:2)	$C_{47}H_{86}O_6K$	$[M+K]^+$	785.6056	18.9
794.7239	TG(46:1)	$C_{49}H_{96}NO_6$	$[M+NH_4]^+$	794.7232	19.2
796.7394	TG(46:0)	$C_{49}H_{98}NO_6$	$[M+NH_4]^+$	796.7389	19.7
807.6359	SM(d18:2/22:0)	$C_{45}H_{89}N_2O_6PNa$	$[M+Na]^+$	807.6350	12.5
809.6508	SM(d18:1/22:0)	$C_{45}H_{91}N_2O_6PNa$	$[M+Na]^+$	809.6507	13.9
813.6846	SM(d18:1/24:1)	$C_{47}H_{94}N_2O_6P$	$[M+H]^+$	813.6844	14.3
815.6999	SM(d18:1/24:0)	$C_{47}H_{96}N_2O_6P$	$[M+H]^+$	815.7000	15.6
820.7393	TG(48:2)	$C_{51}H_{98}NO_6$	$[M+NH_4]^+$	820.7389	19.3
822.7553	TG(48:1)	$C_{51}H_{100}NO_6$	$[M+NH_4]^+$	822.7545	19.7
824.7704	TG(48:0)	$C_{51}H_{102}NO_6$	$[M+NH_4]^+$	824.7702	20.4
833.6508	SM(d42:3)	$C_{47}H_{91}N_2O_6PNa$	$[M+Na]^+$	833.6508	12.5
835.6666	SM(d18:1/24:1)	$C_{47}H_{93}N_2O_6PNa$	$[M+Na]^+$	835.6663	14.3
837.6821	SM(d18:1/24:0)	$C_{47}H_{95}N_2O_6PNa$	$[M+Na]^+$	837.6820	15.6
848.7705	TG(50:2)	$C_{53}H_{102}NO_6$	$[M+NH_4]^+$	848.7702	19.8
850.7862	TG(50:1)	$C_{53}H_{104}NO_6$	$[M+NH_4]^+$	850.7858	20.4
852.8016	TG(50:0)	$C_{53}H_{106}NO_6$	$[M+NH_4]^+$	852.8015	21.1
876.8022	TG(16:0/18:1/18:1)	$C_{55}H_{106}NO_6$	$[M+NH_4]^+$	876.8015	20.5
878.8171	TG(16:0/18:0/18:1)	$C_{55}H_{108}NO_6$	$[M+NH_4]^+$	878.8171	21.1
880.8328	TG(16:0/18:0/18:0)	$C_{55}H_{110}NO_6$	$[M+NH_4]^+$	880.8328	21.9
902.8173	TG(54:3)	$C_{57}H_{108}NO_6$	$[M+NH_4]^+$	902.8171	20.6
904.8331	TG(54:2)	$C_{57}H_{110}NO_6$	$[M+NH_4]^+$	904.8324	21.2

Table S5 High-resolution LC-ESI-MS and LC-ESI-MS/MS in negative ion-mode: Deprotonated molecular ions ([M-H]⁻) as well as formate adducts ([M+HCOO]⁻) of 40 unambiguously identified lipids in methanolic extracts of human aortic tissue slices

m/z	Annotation	Formula	Ion	$m/z_{(calc)}$	t_R
610.5417	Cer(d18:1/18:0)	$C_{37}H_{72}NO_5$	$[M+Formate]^-$	610.5417	11.5
638.5728	Cer(d18:1/20:0)	$C_{39}H_{76}NO_5$	$[M+Formate]^-$	638.5730	13.2
666.6046	Cer(d18:1/22:0)	$C_{41}H_{80}NO_5$	$[M+Formate]^-$	666.6043	14.8
694.6359	Cer(d18:1/24:0)	$C_{43}H_{84}NO_5$	$[M+Formate]^-$	694.6356	16.0
719.5352	SM(d18:1/14:0)	$C_{38}H_{76}N_2O_8P$	$[M+Formate]^-$	719.5345	6.7
733.5505	SM(d18:1/15:0)	$C_{39}H_{78}N_2O_8P$	$[M+Formate]^-$	733.5502	7.4
745.5504	SM(d34:2)	$C_{40}H_{78}N_2O_8P$	$[M+Formate]^-$	745.5502	7.1
747.5658	SM(d18:1/16:0)	$C_{40}H_{80}N_2O_8P$	$[M+Formate]^-$	747.5658	8.1
749.5820	SM(d18:0/16:0)	$C_{40}H_{82}N_2O_8P$	$[M+Formate]^-$	749.5815	8.1
750.5292	PC(16:0/14:0)	$C_{39}H_{77}NO_{10}P$	$[M+Formate]^-$	750.5291	8.4
761.5815	SM(d18:1/17:0)	$C_{41}H_{82}N_2O_8P$	$[M+Formate]^-$	761.5815	9.0
763.6016	SM(d18:0/17:0)	$C_{41}H_{84}N_2O_8P$	$[M+Formate]^-$	763.6016	13.7
773.5815	SM(d18:1/18:1)	$C_{42}H_{84}N_2O_8P$	$[M+Formate]^-$	773.5815	8.5
775.5972	SM(d18:1/18:0)	$C_{42}H_{84}N_2O_8P$	$[M+Formate]^-$	775.5971	9.8
776.5448	PC(16:0/16:1)	$C_{41}H_{79}NO_{10}P$	$[M+Formate]^-$	776.5448	9.8
778.5606	PC(16:0/16:0)	$C_{41}H_{81}NO_{10}P$	$[M+Formate]^-$	778.5604	10.1
801.6130	SM(d38:2)	$C_{44}H_{86}N_2O_8P$	$[M+Formate]^-$	801.6128	10.2
802.5611	PC(16:0/18:2)	$C_{43}H_{81}NO_{10}P$	$[M+Formate]^-$	802.5604	9.2
803.6292	SM(d18:1/20:0)	$C_{44}H_{88}N_2O_8P$	$[M+Formate]^-$	803.6284	11.7
804.5761	PC(16:0/18:1)	$C_{43}H_{83}NO_{10}P$	$[M+Formate]^-$	804.5761	10.3
806.5920	PC(16:0/18:0)	$C_{43}H_{85}NO_{10}P$	$[M+Formate]^-$	806.5917	11.9
817.6448	SM(d18:1/21:0)	$C_{45}H_{90}N_2O_8P$	$[M+Formate]^-$	817.6441	12.5
826.5608	PC(16:0/20:4)	$C_{45}H_{81}NO_{10}P$	$[M+Formate]^-$	826.5604	9.1
829.6443	SM(d18:2/22:0)	$C_{46}H_{90}N_2O_8P$	$[M+Formate]^-$	829.6441	12.0
830.5926	PC(18:0/18:2)	$C_{45}H_{85}NO_{10}P$	$[M+Formate]^-$	830.5917	10.9
831.6597	SM(d18:1/22:0)	$C_{46}H_{92}N_2O_8P$	$[M+Formate]^-$	831.6597	13.3
832.6077	PC(36:1)	$C_{45}H_{87}NO_{10}P$	$[M+Formate]^-$	832.6074	12.2
834.5292	PS(40:6)	$C_{46}H_{77}NO_{10}P$	$[M-H]^-$	834.5291	8.6
835.5346	PI(34:1)	$C_{43}H_{80}O_{13}P$	$[M-H]^-$	835.5342	7.8
836.5452	PS(40:5)	$C_{46}H_{79}NO_{10}P$	$[M-H]^-$	836.5447	9.4
837.5508	PI(34:0)	$C_{43}H_{82}O_{13}P$	$[M-H]^-$	837.5499	9.0

845.6755	SM(d18:1/23:0)	$C_{47}H_{94}N_2O_8P$	$[M+Formate]^-$	845.6754	14.3
854.5925	PC(18:0/20:4)	$C_{47}H_{85}NO_{10}P$	$[M+Formate]^-$	854.5917	10.9
855.6597	SM(d42:3)	$C_{48}H_{92}N_2O_8P$	$[M+Formate]^-$	855.6597	12.0
857.6758	SM(d18:1/24:1)	$C_{48}H_{94}N_2O_8P$	$[M+Formate]^-$	857.6754	13.3
859.6913	SM(d18:1/24:0)	$C_{48}H_{96}N_2O_8P$	$[M+Formate]^-$	859.6910	15.1
861.5485	PI(36:2)	$C_{45}H_{82}O_{13}P$	$[M-H]^-$	861.5499	8.2
863.5664	PI(36:1)	$C_{45}H_{84}O_{13}P$	$[M-H]^-$	863.5655	9.4
872.6835	PC(O-30:2)	$C_{39}H_{75}NO_9P$	$[M+Formate]^-$	872.6833	12.3
885.5496	PI(18:0/20:4)	$C_{47}H_{82}O_{13}P$	$[M-H]^-$	885.5499	8.3

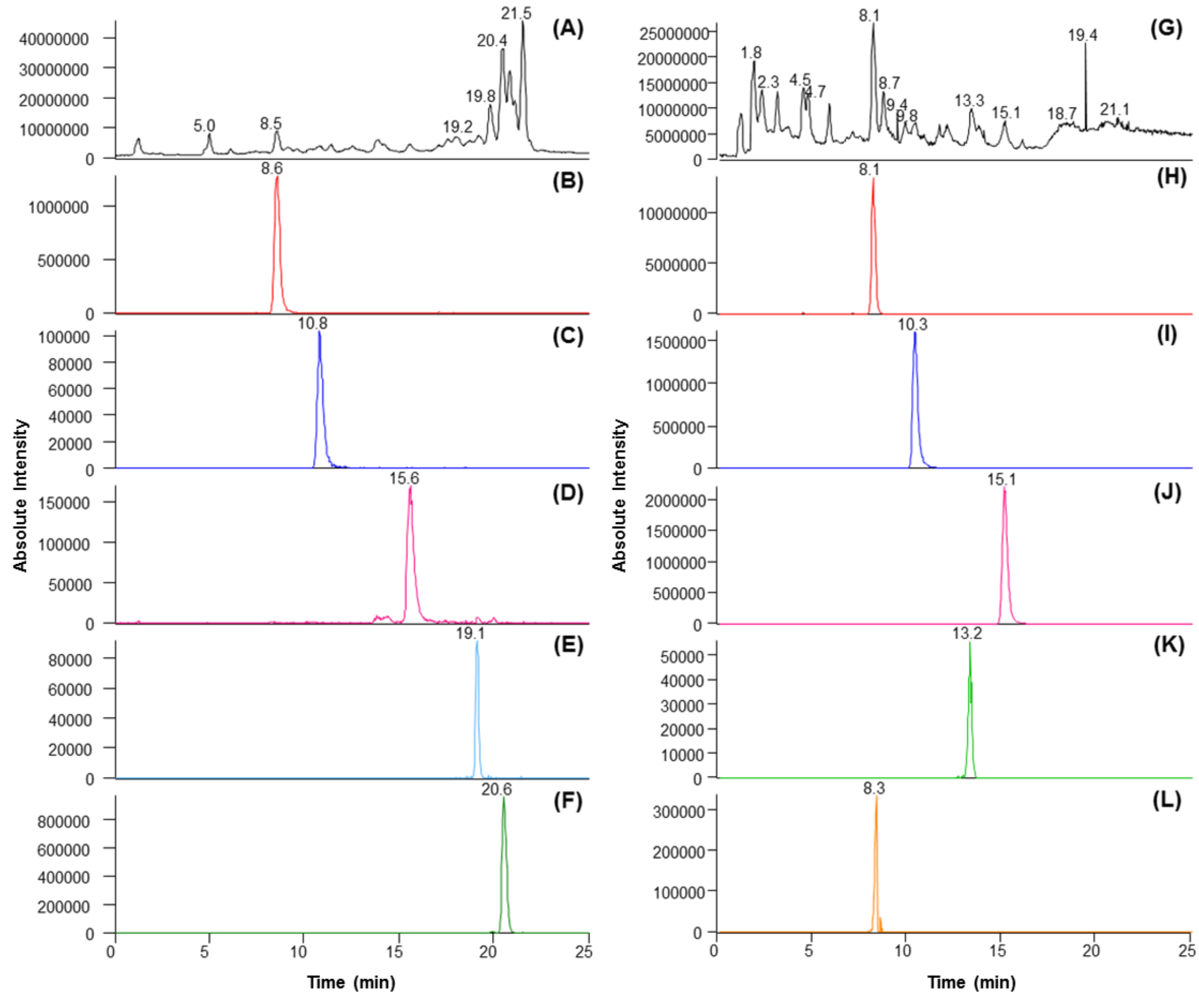

Fig. S11 LC-ESI-MS analyses of extracts of human aortic tissue slices. Left: Positive ion-mode TIC chromatogram (A) and exemplary extracted-ion chromatograms of (B) SM(d18:1/16:0) at m/z 725 ($[M+Na]^+$); (C) PC(16:0/18:1) at m/z 760 ($[M+H]^+$); (D) SM(d18:1/24:0) at m/z 815 ($[M+H]^+$); (E) TG(44:0) at m/z 768 ($[M+NH_4]^+$); (F) TG(54:3) at m/z 902 ($[M+NH_4]^+$); Right: negative ion-mode TIC chromatogram (G) and exemplary extracted-ion chromatograms of (H) SM(d18:1/16:0) at m/z 747 ($[M+Formate]^-$); (I) PC(16:0/18:1) at m/z 804 ($[M+Formate]^-$); (J) SM(d18:1/24:0) at m/z 859 ($[M+Formate]^-$); (K) Cer(d18:1/20:0) at m/z 638 ($[M+Formate]^-$); (L) PI(18:0/20:4) at m/z 885 ($[M-H]^-$). The systematic 0.5 min shift of the retention times (B/H; C/I; D/J) was caused by slightly different backpressures at the two different ESI sources

References

1. Yang JH, Caprioli RM. Matrix Sublimation/Recrystallization for Imaging Proteins by Mass Spectrometry at High Spatial Resolution. Anal Chem. 2011;83(14):5728-34. doi:10.1021/ac200998a.
2. Strohalm M, Hassman M, Kosata B, Kodicek M. mMass data miner: an open source alternative for mass spectrometric data analysis. Rapid Commun Mass Sp. 2008;22(6):905-8. doi:10.1002/rcm.3444.
3. Lerno LA, German JB, Lebrilla CB. Method for the Identification of Lipid Classes Based on Referenced Kendrick Mass Analysis. Anal Chem. 2010;82(10):4236-45. doi:10.1021/ac100556g.
4. Gibb S, Strimmer K. MALDIquant: a versatile R package for the analysis of mass spectrometry data. Bioinformatics. 2012;28(17):2270-1. doi:10.1093/bioinformatics/bts447.
5. Bemis KD, Harry A, Eberlin LS, Ferreira C, van de Ven SM, Mallick P et al. Cardinal: an R package for statistical analysis of mass spectrometry-based imaging experiments. Bioinformatics. 2015;31(14):2418-20. doi:10.1093/bioinformatics/btv146.
6. Kendrick E. A Mass Scale Based on Ch2=14.0000 for High Resolution Mass Spectrometry of Organic Compounds. Anal Chem. 1963;35(13):2146-&. doi:DOI 10.1021/ac60206a048.
7. Hughey CA, Hendrickson CL, Rodgers RP, Marshall AG, Qian KN. Kendrick mass defect spectrum: A compact visual analysis for ultrahigh-resolution broadband mass spectra. Anal Chem. 2001;73(19):4676-81. doi:DOI 10.1021/ac010560w.
8. Doppler C, Arnhard K, Dumfarth J, Heinz K, Messner B, Stern C et al. Metabolomic profiling of ascending thoracic aortic aneurysms and dissections - Implications for pathophysiology and biomarker discovery. PloS one. 2017;12(5):e0176727. doi:10.1371/journal.pone.0176727.
9. Kaufmann R, Spengler B, Lutzenkirchen F. Mass-Spectrometric Sequencing of Linear Peptides by Product-Ion Analysis in a Reflectron Time-of-Flight Mass-Spectrometer Using Matrix-Assisted Laser-Desorption Ionization. Rapid Commun Mass Sp. 1993;7(10):902-10. doi:DOI 10.1002/rcm.1290071010.
10. Sato Y, Bernier F, Suzuki I, Kotani S, Nakagawa M, Oda Y. Comparative lipidomics of mouse brain exposed to enriched environment. J Lipid Res. 2013;54(10):2687-96. doi:10.1194/jlr.M038075.
